# Editorial: Towards the rapid and systematic assessment of vaccine technologies

**DOI:** 10.3389/fimmu.2026.1816160

**Published:** 2026-03-10

**Authors:** Jessica Z. Kubicek-Sutherland, Benjamin McMahon, Evan Skowronski, Robert P. Webb, Traci Pals

**Affiliations:** 1 Los Alamos National Laboratory, Los Alamos, NM, United States; 2 TMG Biosciences, LLC, Conroe, TX, United States; 3 Joint Science and Technology Office, Defense Threat Reduction Agency, Fort Belvoir, VA, United States

**Keywords:** artificial intelligence, computational biology, immunology, negative data, standardization, vaccine development, vaccinology

The COVID-19 pandemic highlighted both the extraordinary potential of modern vaccinology and persistent challenges in how vaccine technologies are assessed. While vaccines can be developed and deployed at unprecedented speed, our ability to predict efficacy in a population is constrained by methodological difficulties, underreporting of negative results, and limited comparability across studies. This editorial introduces a Research Topic that brings together an interdisciplinary collection of experimental, computational, and theoretical contributions spanning multiple pathogens and vaccine platforms. Across these contributions, emerging themes emphasize the need for standardized immunogenicity metrics, transparent reporting including negative findings, and harmonized experimental protocols to support meaningful comparisons. This editorial highlights community practices and shared commitments – supported by researchers, funders, and journals – that could strengthen reproducibility, transparency, and cumulative learning in vaccine research.

## 1 Introduction: lessons learned and the urgency of preparedness

The COVID-19 pandemic demonstrated both the remarkable potential of modern vaccinology and the limits of our preparedness. While vaccines were developed at unprecedented speed, success depended on decades of prior investment, extensive trial and error, and an urgent mobilization that may not be easily replicated in the future (1). Vaccine design, testing, and manufacturing remain resource-intensive and operationally complex. On average it costs over $800 million and takes 10 years to bring a new vaccine to market (2). As the world faces the growing threat of infectious diseases that are emerging and re-emerging, naturally occurring and deliberately engineered, greater coordination across experimental, computational, and policy domains is required. This Research Topic was conceived to explore how advances across diverse disciplines might collectively improve how vaccine technologies are assessed, compared, and advanced under time pressure.

## 2 The challenge: variability, lack of comparability, and the need for standardization

The COVID-19 pandemic has accelerated the global vaccine development process (3). However, the field remains constrained by high methodological variability, where experimental data are generated under diverse conditions. Differences in animal models, immunization schedules, sample types, immunoassays, and analysis pipelines complicate direct comparison across studies. Even when investigating the same pathogen or platform, laboratories have independent criteria for defining successful outcomes, leaving results difficult to reconcile. The outcome of inconsistent methods and reporting is a reduced ability to identify shared patterns of success or failure across vaccine technologies. The lack of comparability in vaccine datasets slows vaccine discovery, increases costs, and complicates decision-making during public health emergencies. Several contributions in this Research Topic explicitly confront this challenge, highlighting how limited comparability can hinder interpretation and slow decision-making during public health emergencies. Multiple studies in this Research Topic emphasize that negative or neutral results are often among the most informative for understanding platform limitations, yet remain underrepresented in the literature.

## 3 Themes and contributions from this Research Topic

This Research Topic brings together an interdisciplinary collection of research spanning experimental, computational, and theoretical approaches to vaccine development. Collectively, these works advance the overarching goal of enhancing comparability, reproducibility, and transparency across the vaccine research field. The issue spans diverse pathogens – including severe acute respiratory syndrome coronavirus 2 (SARS-CoV-2), Ebola virus (EBOV), *Burkholderia pseudomallei*, *Yersinia pestis*, *Mycobacterium avium* subspecies *paratuberculosis* – and a broad range of vaccine platforms including mRNA, DNA, recombinant vesicular stomatitis virus (rVSV), yeast expression systems, nanoparticle formulations, protein subunit vaccines, glycoconjugates, and live attenuated vaccines.

Computational contributions include mechanistic modeling, artificial intelligence (AI)-assisted data extraction, in silico multi-epitope vaccine design, and strategies for data integration and standardization. Experimental papers present optimized protocols for detecting SARS-CoV-2 T-cell responses, high-throughput PepSeq methods for antigen discovery and immune profiling, and human *in vitro* immunization assay platforms that integrate innate and adaptive immunity.

Across these manuscripts, a recurring message emerges: progress depends not only on novel technologies, but also on shared experimental practices, transparent reporting, and community standards that enable results to be compared and reused. Several reviews and methodological studies in this Research Topic highlight persistent barriers to computational integration including inconsistent metadata standards, heterogeneous datasets, and the limited availability of negative results that constrain the effective application of machine learning (ML) in vaccinology. The consensus mathematical model of antibody dynamics directly addresses several of these challenges by providing a generalizable *in-silico* platform for cross-pathogen and cross-platform comparisons. Methodological studies further emphasize the importance of reporting negative and neutral data, both to understand failure modes and to train more representative computational models. This theme resonates across both experimental and computational contributions in this Research Topic, reflecting a community-wide recognition that transparency, including the publication of negative findings, is fundamental to building cumulative knowledge.

## 4 Shared commitments and implications for the field

Taken together, the contributions in this Research Topic suggest a set of shared commitments that could strengthen vaccine research and preparedness. These commitments span multiple stakeholders:

- Researchers: adopting standardized experimental designs where feasible and reporting negative or neutral findings
- Journals: valuing transparency, methodological detail, and the publication of non-positive results
- Funders and sponsors: supporting shared reference standards, data infrastructure, and cross-platform benchmarking efforts

Emphasizing reproducibility, transparency, and comparability across studies would enable faster learning from both successes and failures. Aligning experimental protocols, harmonizing assay endpoints, and systematically capturing both positive and negative outcomes can help transform fragmented studies into a more cumulative and interpretable evidence base. Investments in open repositories, consensus reference standards, and shared benchmarking practices would further support coordination across laboratories and platforms.

This Research Topic demonstrates the breadth of ongoing innovation in vaccine research while underscoring the need for convergence on shared experimental and reporting practices. Advancing pandemic preparedness will depend not only on continued development of new vaccine technologies, but also on sustained investment in data infrastructure, community standards, and collaboration among experimentalists, computational scientists, funders, and policymakers.

## 5 A call to arms

The development of the COVID-19 vaccine was an unprecedented global achievement but should also serve as a warning for the need to establish a cohesive, collaborative response in anticipation of the next potential pandemic-level event. The individual tasks involved in the design, development, assessment, approval, and distribution of vaccines need to be accelerated, but with a systematic and standardized approach so the global health community can adopt a proactive, rather than reactive, response. This Research Topic collectively describes the start of integrated efforts to leverage both published and experimental vaccine study data, assessed under tightly standardized methodology, to support the rapid and systematic assessment of vaccine technologies.

AI and ML offer a powerful, but still under-realized, opportunity to accelerate vaccine research by enabling the integration, comparison, and interpretation of heterogeneous experimental data at scale (4–6). The performance and generalizability of these methods remain fundamentally constrained by fragmented data ecosystems, inconsistent metadata standards, limited access to raw experimental results, and the systematic absence of negative or neutral outcomes from the published record. Without coordinated efforts to standardize, curate, and share vaccine study data across platforms and institutions, AI risks amplifying existing biases rather than delivering transformative insight. Addressing these limitations will require sustained community investment in interoperable data infrastructures, transparent reporting practices, and data sharing. To translate these priorities into action, we propose the following next steps for the field:

- Standardize metadata frameworks across vaccine studies to improve cross-study analysis.
- Invest in shared data infrastructures that enable secure and responsible data exchange and cross-institution collaboration.
- Adopt transparent reporting practices that include comprehensive documentation of experimental design, reagent sourcing, data pre-processing procedures, and analytical workflows.
- Actively publish or deposit negative and non-positive findings in accessible repositories to reduce publication bias, improve analytical robustness, and build a more representative evidence base.
- Partner across research groups to responsibly integrate well-annotated datasets, including those not yet publicly available.

Collective commitment to these priorities will be necessary to build a more transparent, representative, and durable foundation for AI-enabled vaccinology.
